# Empirical leucine-to-carbon conversion factors in north-eastern Atlantic waters (50–2000 m) shaped by bacterial community composition and optical signature of DOM

**DOI:** 10.1038/s41598-021-03790-y

**Published:** 2021-12-21

**Authors:** C. Pamela Orta-Ponce, Tamara Rodríguez-Ramos, Mar Nieto-Cid, Eva Teira, Elisa Guerrero-Feijóo, Antonio Bode, Marta M. Varela

**Affiliations:** 1 Instituto Español de Oceanografía (IEO-CSIC), Centro Oceanográfico de A Coruña, 15080 A Coruña, Spain; 2 Facultade de Ciencias, Zapateira, Universidade da Coruña, 15071 A Coruña, Spain; 3 Laboratorio de Geoquímica Orgánica, Instituto de Investigaciones Marinas (CSIC), 36208 Vigo, Spain; 4 Departamento de Ecología y Biología Animal, Universidade de Vigo, Centro de Investigación Mariña da Universidade de Vigo (CIM-UVigo), 36310 Vigo, Spain

**Keywords:** Carbon cycle, Microbial biooceanography, Marine microbiology, Metagenomics

## Abstract

Microbial heterotrophic activity is a major process regulating the flux of dissolved organic matter (DOM) in the ocean, while the characteristics of this DOM strongly influence its microbial utilization and fate in the ocean. In order to broaden the vertical resolution of leucine-to-carbon conversion factors (CFs), needed for converting substrate incorporation into biomass production by heterotrophic bacteria, 20 dilution experiments were performed in the North Atlantic Ocean. We found a depth-stratification in empirical CFs values from epipelagic to bathypelagic waters (4.00 ± 1.09 to 0.10 ± 0.00 kg C mol Leu^−1^). Our results demonstrated that the customarily used theoretical CF of 1.55 kg C mol Leu^−1^ in oceanic samples can lead to an underestimation of prokaryotic heterotrophic production in epi- and mesopelagic waters, while it can overestimate it in the bathypelagic ocean. Pearson correlations showed that CFs were related not only to hydrographic variables such as temperature, but also to specific phylogenetic groups and DOM quality and quantity indices. Furthermore, a multiple linear regression model predicting CFs from relatively simple hydrographic and optical spectroscopic measurements was attempted. Taken together, our results suggest that differences in CFs throughout the water column are significantly connected to DOM, and also reflect differences linked to specific prokaryotic groups.

## Introduction

Heterotrophic bacteria are key in the cycling of dissolved organic matter (DOM) because they are major consumers and transformers of the DOM pool in the ocean^1^, which in turn supports the metabolic activities and growth of bacterioplankton^2^. Thus, prokaryotic heterotrophic production (PHP) is a strategic variable for evaluating the relevance of heterotrophic bacterioplankton in the ocean carbon cycling^1,3,4^. However, PHP cannot be measured directly and is rather estimated from related metabolic processes. The most widespread method for PHP estimation, due to its high sensitivity and reduced incubation time, is the measurement of the incorporation rate of radiolabeled amino acids, such as ^3^H-leucine^5^. From leucine incorporation rates (LIR), PHP can be transformed into carbon units by using a conversion factor (CF)^6^. A theoretical CF of 1.55 kg C mol Leu^−1^, assuming no isotope dilution, and based on average protein and carbon content of bacterial cells, has been traditionally used for ocean waters^6^. However, while the relation between substrate incorporation and carbon produced is variable^7^, this theoretical CF is a constant. Hence, the variability of protein and carbon content in bacterial cells growing in different environments (e.g., coastal vs. oceanic systems, and/or surface vs. deep waters) is not accounted for, potentially leading to misinterpretation of the resultant PHP estimates. Hitherto, most of the experiments that have been developed to determine in situ empirical CFs (eCFs) were carried out in epipelagic, open-ocean waters^3,8–10^. Those eCFs were found to be highly variable, both seasonally^9^ and spatially, throughout the global ocean^3^. Most importantly, marine microbiologists deal with the limited availability of eCFs estimates from deep marine ecosystems: there are only a few studies in mesopelagic waters^11,12^, while no eCFs are available for bathypelagic waters. Additionally, there is lack of studies concurrently measuring eCFs, DOM and the taxonomic composition of the microbial community allowing to examine the link among these variables.

Depth-dependent biogeographical patterns of CFs, and in turn PHP, are likely dependent on both the availability of substrates for prokaryotic activity as well as on the capability of certain microorganisms to use those resources depending on their nature and quality^13^. Therefore, the composition of the DOM pool may have a role in shaping bacterial community structure and vice versa^14,15^, and these factors likely influence PHP, as well. However, the relationship between microorganisms diversity and DOM is still poorly understood and, consequently, their impact on CFs, and ultimately on the ocean carbon cycle.

DOM can be characterized by its optical properties: on the one hand, part of the DOM absorbs light and constitutes the chromophoric DOM (CDOM); on the other, the fluorescent DOM (FDOM) is the fraction of the CDOM which emits fluorescence when it is irradiated^16,17^. The CDOM absorption coefficients at 254 nm (a254), 340 nm (a340) and 365 nm (a365) provide information about the reactivity/complexity of molecules within the DOM pool^15^. Albeit the ecological significance of these indices is still unclear, the DOM conjugation/aromaticity increases with the wavelength^17^, so that absorption coefficients at wavelengths higher than 300 nm would gather information related to molecules more complex/aromatic^15^. In addition, the spectral slope between 275 and 295 nm (s275-295), a proxy of DOM molecular weight, has been used to relate the optical properties of DOM with marine microorganisms and its biological bioreactivity^16^. FDOM measurements at specific excitation/emission wavelength pairs also provide information on humic-like marine substances (peak M, refractory DOM resistant to microbial degradation) and protein-like molecules (peak T, freshly-produced labile DOM)^15,18^. From previous studies, it has been suggested that bacterial community structure is vertically stratified and that these patterns are linked to DOM optical properties^4,15,19^.

In this work, vertical variability of the eCFs was concomitantly studied with bacterial diversity and the optical signature of DOM at two stations near Cape Finisterre (North Atlantic Ocean off Galicia; NW Iberian Peninsula) and Santander (Bay of Biscay). The NW Iberian upwelling system off the Galician coast is a very dynamic area characterized by seasonal upwelling pulses of variable annual intensity^20^, which support both offshore export and sinking fluxes of organic matter^21^. Mixing of different water masses reaches down to the mesopelagic layer that flows northwards along the western Iberian Peninsula^22^. Comparatively, in the Santander section, in the eastern limit of the upwelling region, the upwelling events are usually shorter and reach lower intensities than off the Galician coast^23^.

The main objectives of this work are: (1) to empirically determine in situ eCFs for different depth layers: epipelagic (< 100 m), upper mesopelagic (100–450 m), lower mesopelagic (450–1000 m) and bathypelagic (> 1000 m) waters, in the North Atlantic Ocean, and (2) to explore the potential relationship of eCFs with the optical signature of DOM and bacterial community composition.

## Results

### Hydrographic characterization of the study area

**Figure 1:**
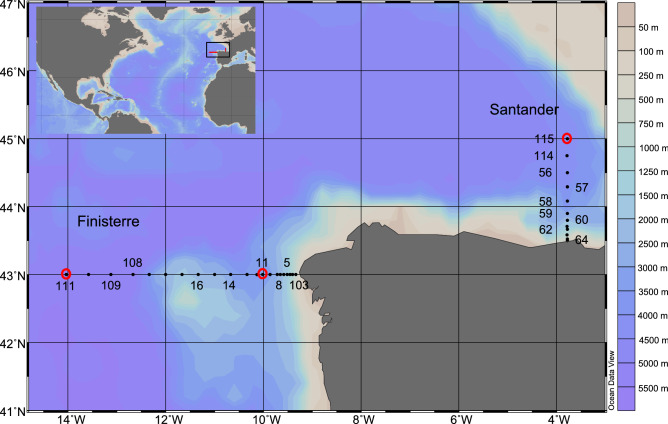
Location of the sampling stations along Finisterre and Santander sections during the MODUPLAN 0814 cruise. Black dots indicate the stations in which hydrographic features of the water column were measured (see Supplementary Information, Fig. S1). The numbers indicate the stations in which leucine incorporation rate (LIR) was measured. The experiments to determine empirical leucine-to-carbon conversion factors (eCFs) were carried out at biological stations 11, 111 and 115 (highlighted with a red circle). Bacterial diversity and the quality and quantity of DOM were determined at stations 11 and 115.

The location of the sampling stations across both sections (Finisterre and Santander) is shown in Fig. 1. At each station, the hydrographic properties found throughout the water column (Table 1 and Supplementary Information, Fig. S1) were used to select the sampling depths (see Experimental procedures). Hydrographic conditions were similar in both sections, particularly in the epipelagic and upper mesopelagic layers. In both sections, lower mesopelagic waters showed the minimum oxygen concentration (Oxy) at ~ 950 m related to the signal of the Mediterranean water, characterized by high salinity (Sal), particularly apparent in Finisterre (Table 1). In the bathypelagic layer, the lowest temperature and salinity, and relatively high mean dissolved oxygen concentration were recorded (Table 1).

**Table 1:** Range of vertical variability of the hydrographic features measured along the Finisterre and Santander sections (north-east Atlantic Ocean): potential temperature (Tpot), salinity (Sal) and dissolved oxygen concentration (Oxy).

Depth layer	Depth (m)	Finisterre			Santander		
		Tpot (°C)	Sal	Oxy (µmol kg^−1^)	Tpot (°C)	Sal	Oxy (µmol kg^−1^)
epipelagic	< 100	12.31–20.86	35.54–35.87	215.67–299.42	11.92–21.82	34.68–35.63	207.25–285.64
upper mesopelagic	100–450	11.10–13.55	35.60–35.81	198.74–248.75	10.91–12.31	35.60–35.64	205.96–247.00
lower mesopelagic	450–1000	10.06–11.46	35.59–36.12	178.44–228.41	9.61–11.36	35.60–35.79	183.90–221.31
bathypelagic	> 1000	2.30–11.13	34.90–36.19	179.22–259.89	2.10–9.92	34.90–35.80	184.40–249.56

### Empirical leucine-to-carbon CFs and derived PHP throughout the water column

The values of the eCFs decreased with depth in both sections (Table 2), ranging from 4.00 to 0.65 in Finisterre and from 0.43 to 0.10 kg C mol Leu^−1^ in Santander. Importantly, eCFs determined in epi- and mesopelagic waters of Finisterre, were higher than the theoretical CF (1.55 kg C mol Leu^−1^), except for the lower mean eCF value (1.20 kg C mol Leu^−1^) measured at 1000 m depth sample in station 11 (Table 2). Conversely, eCFs in the bathypelagic waters were often lower than the theoretical CF particularly in Santander. Additionally, it is important to highlight that eCFs were significantly different between Finisterre and Santander sections (Student’s t-test, *P* < 0.05), and among depths for the Finisterre section (ANOVA, *P* < 0.5). Consequently, estimating eCFs at different depths and locations is crucial for understanding PHP trends.

**Table 2:** Empirical carbon conversion factors (eCFs, kg C mol Leu^−1^) determined in stations 111, 11 and 115 of Finisterre (FIN) and Santander (SAN) sections, respectively.

sect	st	Depth range	Depth (m)	eCF (± se)^a^ (kg C mol Leu^−1^)	P^a^	n^b^	eCF (± se)^b^ (kg C mol Leu^−1^)	eCF (± se)^c^ (kg C mol Leu^−1^)
FIN	111	epipelagic	50	3.91 (± 0.45)	0.0003	1	3.91 (± 0.45)	3.91 (± 0.45)
		Upper mesopelagic	100	4.00 (± 1.09)	0.0573	1	4.00 (± 1.09)	4.00 (± 1.09)
FIN	11	Lower mesopelagic	500	2.12 (± 0.43)	0.0378	1	2.12 (± 0.43)	2.12 (± 0.43)
		Lower mesopelagic	1000_a	1.58 (± 0.36)	0.0473	3	1.19 **(**± 0.22**)**	1.39 (± 0.21)
				0.96 (± 0.15)	0.0033			
				1.03 (± 0.15)	0.0020			
		Lower mesopelagic	1000_b	0.78 (± 0.16)	0.0340	2	1.59 **(**± 0.20**)**	
				2.40 (± 0.24)	0.0020			
				0.87(± 0.65)*	0.2705*			
		bathypelagic	2000	1.78 (± 0.33)	0.0057	3	1.50 (± 0.25)	1.50 (± 0.25)
				0.65 (± 0.27)	0.0515			
				2.08 (± 0.75)	0.0512			
SAN	115	Lower mesopelagic	500	0.43 (± 0.13)	0.0260	3	0.31 (± 0.07)	0.31 (± 0.07)
				0.30 (± 0.05)	0.0040			
				0.21 (± 0.03)	0.0039			
		Lower mesopelagic	1000	0.43 (± 0.09)	0.0057	3	0.31 (± 0.08)	0.31 (± 0.08)
				0.36 (± 0.11)	0.0305			
				0.14 (± 0.04)	0.0195			
		bathypelagic	2000	0.11 (± 0.04)	0.0483	2	0.10 (± 0.02)	0.10 (± 0.02)
				0.09 (± 0.00)	0.0075			

Significant values are in bold.

^a^Values of eCF and *P* value per experiment curve. Significant experiments (*P* value threshold, *P* < 0.1) were considered for calculating mean eCF values per sample.

^b^Number of experiments and mean eCF values per sample.

^c^Final eCF values per depth.

*This experiment curve was not used in this study as the regression was not significant.

**Figure 2:**
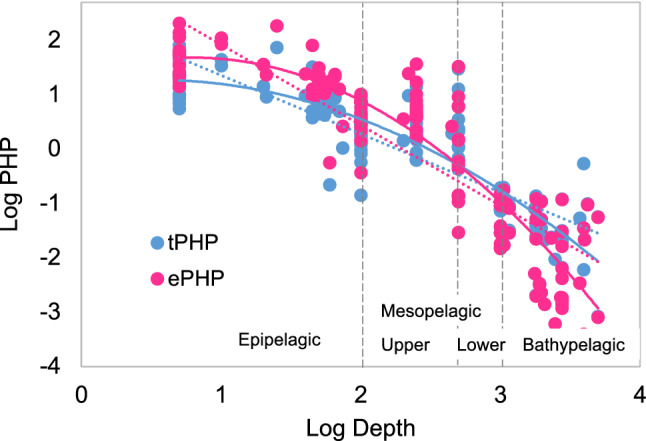
Log–log relationship between depth (m) and PHP (μmol C m^−3^ d^−1^), derived from empirical and theoretical conversion factors (ePHP in fuchsia and tPHP in light blue, respectively). Dashed and solid lines represent, respectively, linear and quadratic models fitted to data. Akaike’s Information Criterion (AIC, in brackets) was used for the selection of the best fitted model (lower AIC). For theoretical estimates, the linear model was y = − 1.08x + 2.43 (R^2^ = 0.75) [AIC = 232.11], and the quadratic model was y = -0.33x^2^ + 0.35x + 1.19 (R^2^ = 0.80) [AIC = 200.80]; for empirical PHP, the fitted linear model was y = 1.48x + 3.40 (R^2^ = 0.76) [AIC = 311.05], and the quadratic model was y = 0.55x^2^ + 0.88x + 1.33 (R^2^ = 0.84) [AIC = 258.84].

PHP (Supplementary Information, Fig. S2) mirrored the variability of LIR and CFs with depth. The theoretical PHP (tPHP) displayed a quadratic (log–log) relationship with depth (Fig. 2). Because tPHP is a linear function of LIR multiplied by the constant theoretical CF (see Methods), log LIR versus log depth would also fit to a quadratic curve (data not shown). Maximum values of empirical PHP (ePHP) were found in the epipelagic and upper mesopelagic layers in both sections, being > twofold higher than tPHP. However, for the lower mesopelagic waters ePHP was ~ twofold higher in Finisterre but ~ fivefold lower in Santander compared to tPHP estimates. Finally, at the bathypelagic layer the constant theoretical CF overestimated PHP in Santander, while there were no significant differences in Finisterre between ePHP and tPHP. Hence, a stronger vertical gradient emerged for ePHP compared to tPHP at the Santander section (Supplementary Information, Fig. S2). The depth-dependence of ePHP was also best resembled by a quadratic than by a linear model (Fig. 2), i.e., the slope of PHP versus depth varied with depth. There were no significant differences between averaged empirical and theoretical PHP of Finisterre and Santander sections (Student-t test, *P* > 0.1). However, significant differences were found for the average ePHP at different depth ranges (One-way ANOVA, *P* < 0.0001), with epipelagic ePHP significantly higher than upper and lower mesopelagic, and bathypelagic ePHP (Tukey’s post hoc test, *P* < 0.001) in both sections. The upper and lower mesopelagic, and bathypelagic samples were not significantly different from each other (Tukey’s post hoc test, *P* > 0.1) in Santander, however, significant differences among meso- and bathypelagic waters were found in Finisterre (Tukey’s post hoc test, *P* < 0.01).

### Vertical variability in bacterial diversity and community composition

In this study, due to the low contribution of archaea (~ 20%, see^19^) to total prokaryotic abundance, we assumed that most of the LIR was carried out by bacteria. Consequently, only bacterial diversity is considered in the following analyses.

After rarefaction, a total of 91,182 reads were classified into 1,055 and 1,499 amplicon sequence variants (ASVs) at stations 11 (Finisterre) and 115 (Santander), respectively. Bacterial diversity showed some geographic differences, with the mean value of the Shannon’s index (H') lower in Finisterre (mean ± sem: 4.26 ± 0.21) than in Santander (4.69 ± 0.21) (Fig. S3, and Supplementary Information Table S1). On the contrary, both H’ and the estimated ASV richness (SChao_1_) did not show a clear vertical pattern (Fig. S3).

The top 37 abundant ASVs/phylotypes (relative abundance > 1%; Fig. S3), with relatively similar vertical distributions at both stations, showed different trends among them. The ASVs belonging to SAR324, SAR202, and the group JL-ETNP-F27 were almost absent within epi- and upper mesopelagic waters, increasing their relative contribution with depth (accounting together for up to 70% and 49% of the total reads in Finisterre and Santander in bathypelagic waters). Other ASVs/phylotypes, such as Actinobacteria, Gammaproteobacteria (such as SAR86, SUP05_2 and Gammaproteobacteria_Others) showed the opposite trend. Other phylotypes, such as SAR406 and those belonging to Alphaproteobacteria (green tones, Fig. S4) did not show a clear trend throughout the water column. Interestingly, a few groups showed opposite patterns between stations. For instance, Planctomycetes were notably abundant at 250, 500 and 2750 m in Finisterre, while this phylum was hardly ever present in mesopelagic waters but it was an important member of bathypelagic communities in Santander. Finally, both stations displayed a mean relative contribution of the group Others (composed of ASVs in very low abundance, accounting for < 1% of total number of reads even after adding them up at Phylum level), higher in bathy- and lower mesopelagic waters than in epi- and upper mesopelagic waters.

In general, the composition of the bacterial community was similar for both stations (ANOSIM, r = 0.22, *P* = 0.21). However, significant differences arose among epi-, upper and lower-mesopelagic, and bathypelagic bacterial communities (ANOSIM, r = 0.57, *P* = 0.02). Among the 37 abundant ASVs/phylotypes*,* Actinomarina_1 and SAR202_Others were the main responsible for the dissimilarities found among communities inhabiting those different depth layers (Supplementary Information Table S2). Actinomarina_1 SAR202_Others, SAR324 (Marine group)_Others and SAR406 contributed to 40% of dissimilarity between epi-, meso- and bathypelagic communities while SAR202_1, SAR202_2 and SAR202_3 accounted for 20% of the dissimilarity between upper and lower mesopelagic waters.

### Vertical variability of DOM

**Figure 3:**
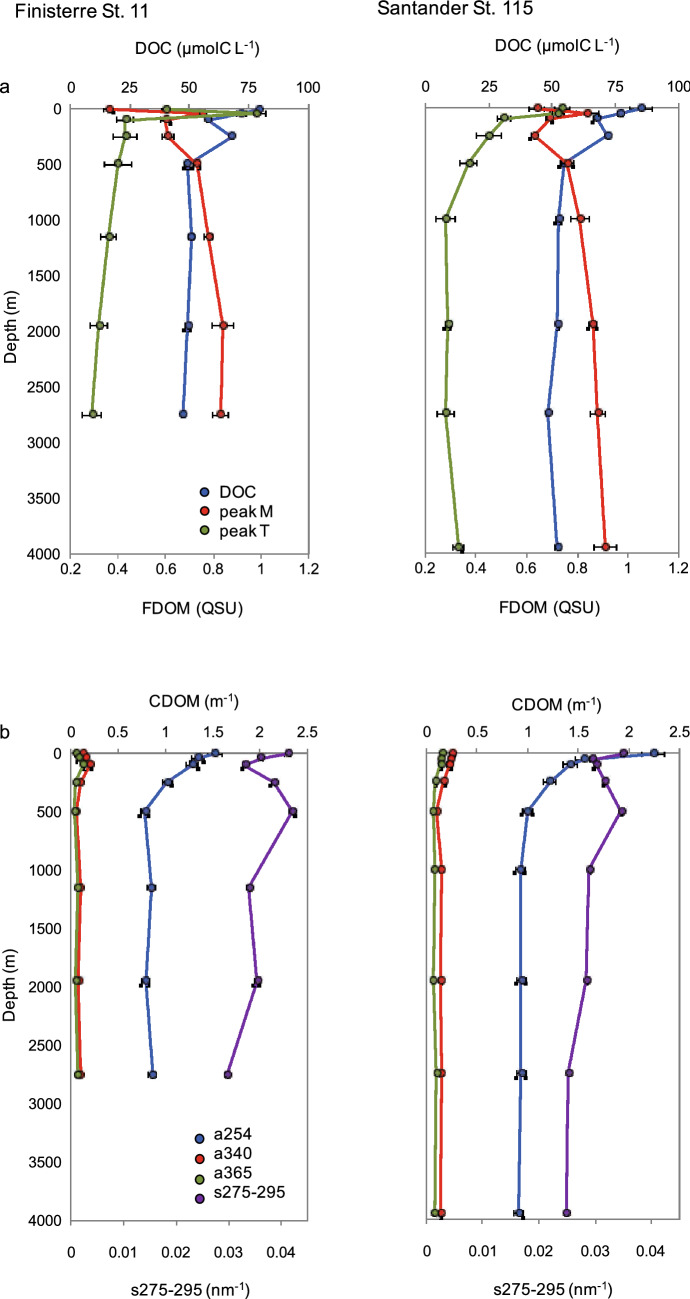
Vertical profiles of: (**a**) dissolved organic carbon (DOC, blue), and fluorescence peaks: peak M (red) and peak T (green); (**b**) absorption coefficients at 254 nm (a254, blue), 340 nm (a340, red) and 365 nm (a365, green), and the absorption slope s275-295 (violet), for Finisterre (left) and Santander (right) sections.

Overall, DOM optical indices showed slightly higher values in Santander than in Finisterre, especially in the epipelagic layer (Fig. 3). Besides, they showed greater variability in epi- and upper mesopelagic waters, while the profiles were much more uniform throughout lower meso- and bathypelagic waters. Dissolved organic carbon (DOC) and DOM optical indices, with the only exception of peak M, decreased with depth (Fig. 3A). Both stations showed maximum values of DOC (mean ± sem: 72.7 ± 3.6 and 81.0 ± 4.1 µmol C L^−1^ for Finisterre and Santander, respectively) and peak T (0.76 ± 0.11 and 0.73 ± 0.03 QSU, respectively) at the epi-pelagic layer, while the minimum values (50.0 ± 0.8 µmol C L^−1^ for DOC and 0.31 ± 0.01 QSU for peak T in both stations) were found in bathypelagic waters (Fig. 3A). Conversely, peak M increased with depth in both stations (from 0.36 to 0.84 ± 0.05 QSU in Finisterre; and from 0.63 to 0.91 ± 0.04 QSU in Santander) (Fig. 3A). The DOM absorption coefficient at 254 nm (a254) decreased exponentially with depth, ranging from 1.48 ± 0.07 m^−1^ to 0.83 ± 0.02 m^−1^ in Finisterre, and from 1.90 ± 0.35 m^−1^ to 0.93 ± 0.01 m^−1^ in Santander (Fig. 3B). Absorption coefficients at 340 nm (a340) and 365 nm (a365) showed very similar vertical trends in both stations, except for epipelagic values. Finally, s275-295 displayed higher vertical variability in the upper 1000 m (Fig. 3B).

### Relationships between eCFs and hydrography, DOM and bacterial diversity

**Figure 4:**
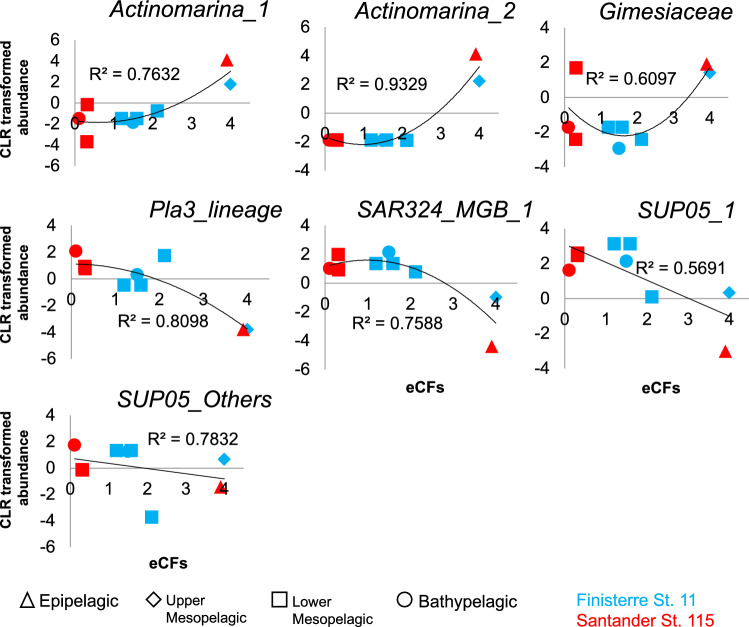
Significant relationships (*P* ≤ 0.05) between the CLR transformed abundance of ASVs/phylotypes and the values of eCFs for the corresponding depth range (triangle, epipelagic; diamond, upper mesopelagic; square, lower mesopelagic; circle, bathypelagic), in stations 11 (Finisterre, blue) and 115 (Santander, red). The black line represents the best quadratic model fitted to data, according to the lowest Akaike’s information criteria (see Table S3 in Supplementary Information). R^2^ is the coefficient of determination of the fitted model.

Significant bivariate correlations were found between eCFs and potential temperature (Tpot) and DOM properties (Table 3). Positive correlations were found with peak T and a254, and negative correlations with peak M, and the ratios of peak M with peak T, DOC and 254 (Table 3). On the other hand, eCFs did not significantly correlate with estimated ASVs richness (SChao_1_) nor with H’. However, they were significantly (*P* ≤ 0.05) related to the centered log-ratio (CLR) transformed abundance of some specific ASVs/phylotypes (Fig. 4). For instance, for the ASVs Actinomarina_1 and Actinomarina_2, and Gimesiaceae, their CLR transformed abundances were best described by a positive quadratic model, decreasing for eCFs values < 2 but increasing for eCFs > 2. Oppositely, the CLR transformed abundance of Pla3_lineage and SAR324_MGB_1 followed a negative quadratic function of eCFs, increasing until eCF ~ 2 to then decrease. In general, these specific taxa greatly determined the observed variations in CFs (R^2^ > 0.5, *P* ≤ 0.05) (Supplementary Information, Table S3).

**Table 3:** Pearson correlation coefficient (r) for the relationship between eCFs and hydrographic features (potential temperature: Tpot; salinity: Sal; dissolved oxygen concentration: Oxy), DOM (dissolved organic carbon concentration: DOC; fluorescence peaks: peak M, peak T; absorption coefficients at 254, 340 and 365 nm: a254, a340, a365; spectral slope between 275 and 295 nm: s275-295) and diversity metrics (ASVs richness estimator: SChao_1_, and Shannon diversity index: H').

Variable group	r	*P*
**Hydrographic features**		
Tpot	**0.65**	0.08
Sal	0.32	0.44
Oxy	0.49	0.22
**DOM properties**		
DOC	0.62	0.10
peak M	− **0.85**	0.01
peak T	**0.66**	0.08
a254	0.63	0.10
a340	0.38	0.36
a365	0.45	0.27
s275-295	0.20	0.64
peak M/peak T	− **0.86**	0.01
peak M/a254	− **0.74**	0.04
peak M/DOC	− **0.83**	0.01
**Bacterial diversity**		
SChao_1_	− 0.01	0.99
H' (Shannon)	− 0.05	0.90

Significant correlations (*P* < 0.1) are highlighted in bold.

Notwithstanding the limited number of samples, and after testing all possible combinations of our variables (including both abundance of specific microbial taxa and DOM composition indices), we obtained a preliminary multiple linear regression model to empirically estimate a CF from temperature and DOM humic and protein fluorescence values (R^2^ = 0.96; *P* = 0.01; n = 8):

$${\text{CF}} = - \left( {0.2 \pm 0.1} \right)\,{\text{Tpot}} - \left( {16 \pm 3} \right)\,{\text{peak}}\,{\text{M}} + \left( {8 \pm 2} \right)\,{\text{peak}}\,{\text{T}} + \left( {13 \pm 3} \right)$$

with *P* = 0.05 for temperature, *P* = 0.01 for peak M and peak T, and *P* = 0.03 for the intercept.

## Discussion

A higher spatial resolution of eCF values is required for an accurate estimation of PHP throughout the water column. To the best of our knowledge, there are no previous studies estimating empirical leucine-to-carbon conversion factors in bathypelagic waters (> 1000 m), and there are very few studies that have investigated their relationship with bacterial diversity^3,10^. Importantly, none of them has investigated the relationship among CFs, bacterial diversity and composition of the DOM pool. A great variability of eCF values (0.09–1.47 kg C mol Leu^−1^) was previously found at epipelagic waters across the world’s open oceans^3^. The eCF values obtained in this study were comparable to those found in other coastal and epipelagic waters, such as at the oligotrophic Mediterranean Sea during summer stratification (0.29–3.25 kg C mol Leu^−1^)^24^, the Galician coast (0.14–3.55 kg C mol Leu^−1^)^8,25–27^, or along an environmental gradient in an estuarine system at the northern South China Sea (0.48–1.69 kg C mol Leu^−1^)^28^.

Overall, the eCFs measured in this study (Table 2) were generally higher than those previously reported for the same depth range (mean ± sem: 1.16 ± 0.61 kg C mol Leu^−1^ ^11^ and 0.55 ± 0.12 kg C mol Leu^−1^ ^12^). This fact might be likely related to the relatively higher availability of organic substrates in our area of study^21,29–31^, especially in Finisterre^32^, where eCFs were remarkably higher than in Santander for each depth range.

We found the highest eCF values from surface down to 500 m in Finisterre section, which is consistent with the higher DOC concentrations measured in epipelagic waters of our study area (Fig. S4) compared to epipelagic samples from subtropical north Atlantic waters (54–79 µmol C L^−1^, < 200 m)^33,34^. Indeed, high concentrations of labile DOM accumulated in the epipelagic layer during the upwelling season^18^, representing 50% of the total dissolved organic carbon susceptible of microbial utilization^35^, asserting the key contribution of dissolved organic matter (DOM) to the export of new primary production in the NW Iberian upwelling system. Eventually, this DOM excess produced during the upwelling season support both the offshore export and sinking fluxes of organic matter^34,36,37^. In fact, our results show that deeper down, at 1000 m, the eCF remained relatively high in Finisterre. By contrast, eCFs decreased considerably compared to the theoretical CF in Santander. This circumstance could be partially explained by the biogeochemical differences in water masses among Santander and Finisterre stations. At ~ 1000 m, we found the high-salinity, low oxygen signature of the Mediterranean water (Table 1), with lower DOC concentrations than the water mass immediately above (the North Atlantic Central water, 250–900 m)^15^. In the mesopelagic waters (Mediterranean Water, 1000 m depth and Labrador Sea Water, 2000 m depth) of the Finisterre section it has been shown an intense water mass mixing^22^, resulting in a vertical DOM movement, which support higher activity of bacterial communities compared to Santander^19^. Overall, at the bathypelagic waters of both sections, eCFs estimated in this study were low and similar to those reported in oligotrophic areas^11,12^ and in bathypelagic waters from subtropical north Atlantic waters with similar DOC concentrations (44.07 ± 2.0 µmol C L^−1^, > 2000 m)^32^.

In such a context, we must also take into account that the differences among other CFs estimated in epi- and mesopelagic waters^11,12^ and our study may be influenced by methodological differences in the experimental design. We conducted the manipulation experiments by diluting samples, while Gasol et al*.*^11^ diluted and filtered, and Baltar et al.^12^*.* only filtered. Thus, Gasol et al*.*^11^ and Baltar et al*.*^12^, by filtering the community throughout 0.6 µm, may have left out organic matter aggregates, fact that could explain why they reported lower values than those found in our study. Then, eCFs appear to be lower when using a combination of filtration and dilution, or only filtration, than when using just dilution. This is particularly relevant in our area of study since the North Atlantic coast is strongly affected by upwelling events, particularly in Finisterre, transporting organic matter from the coast to open-ocean^38^ where large aggregates might likely be abundant.

Our results have crucial implications for PHP estimation. Overall, this study has demonstrated that the systematic use of the theoretical CF (1.55 kg C mol Leu^−1^)^6^ would cause an important underestimation of the PHP in epi- and upper mesopelagic waters, but a significant overestimation at the bathypelagic layer, particularly in the Santander section. This result implies the existence of a much more intense gradient of PHP throughout the water column than previously reported. More importantly, our results showed that PHP does not vary linearly with depth but its depth-dependence is best described by a quadratic function. Consequently, in epipelagic waters (where most of the previous studies were carried out) and bathypelagic waters, PHP values were found to be lower than those expected under a linear depth-dependence, while the opposite occurs at the mesopelagic layer. This curvature suggests differences in hydrographic and/or physiological constraints operating throughout the water column. In oligotrophic environments (and extensively in bathypelagic waters, with relatively low availability of organic bio-labile substrates), low eCFs are attributed to energy consumption addressed to preserve metabolic processes rather than producing bacterial biomass (low PHP), so that leucine respiration is essentially destined to maintain the cells alive^28,39^. Conversely, in epi- and mesopelagic waters, our results suggest that the quantity and quality of organic substrates does not limit bacterial biomass production. Thus, leucine incorporation could be mainly destined towards biomass production (relatively higher eCFs and PHP) in comparison to other open-ocean areas or deep waters.

Assuming that the patterns found for this region can be applied to similar areas such as those under the influence of upwelling, the differences between empirical and theoretical PHP calculated (Supplementary Information, Fig. S2) would greatly influence the estimated carbon fluxes mediated by heterotrophic bacterioplankton activity in the ocean. This outcome implies that our current predictions on the role of bacterial remineralization throughout the water column, and hence, on carbon fluxes between surface and the deep ocean, need to be revised. Our results might likely help to reconcile the discrepancy among the amount of carbon sinking out of the surface ocean and the biological carbon demand in the dark ocean^40,41^ depending on location and depth of the study area.

Importantly, we studied the influence of community composition and DOM properties over eCF values. Overall, we did not find a correlation between eCFs and ASV richness (SChao_1_) or the Shannon diversity index. However, those indices may not reflect whether specific microbial taxa are relevant in the degradation of marine DOM^13^. Nevertheless, our study displayed shifts in eCF values which might be partially related to several specific groups. On the one hand, Actinomarina_1, Actinomarina_2, and Gimesiaceae showed positive quadratic relationships with eCFs. Our results suggest that these groups may play a key role in determining prokaryotic activity, particularly in epipelagic waters. Overall, the higher eCFs found at epipelagic waters could be attributed to the occurrence of these abundant bacterial phylotypes stimulated by higher amounts of phytoplankton exudates and/or also higher temperature (Table 1). On the other hand, Pla3_lineage, and SUP05_Others showed high fitted relationships between their average abundance and eCFs, explaining 81% and 78% of their variability, respectively. These phylotypes followed a negative, quadratic function with eCFs, which predicts intermediate eCFs when these bacterial groups are abundant. These groups were particularly abundant in lower mesopelagic waters, which suggest that they might be involved in the degradation of relatively recalcitrant compounds, predominant in these waters. In the same way, SAR324 and SAR202 (Chloroflexi) which were the most abundant groups in both sections at lower mesopelagic and bathypelagic waters, and show strong and weak correlations with CFs, respectively, should be related to the oxidation of recalcitrant dissolved organic matter^42^ and consequently lower eCFs.

For the first time, our results have also shown that eCFs are shaped not only by depth-related hydrographic features and some specific taxa, but also, to a higher extent, by DOM composition. In such a context, peak M revealed as the most relevant variable, with a negative correlation with eCFs (Table 3). Thus, the humic-like substances (more reworked/refractory DOM generated as by-products of respiration processes^18^, i.e., less bioavailable material) were inversely associated with eCFs, producing lower eCF values when DOM is less labile (i.e., bathypelagic waters). Peak T (related to the production of biolabile DOM) and a254 (which is related to DOC^33,43^ and thus considered a quantity factor) were also positive and significantly related to our eCFs, which would indicate that CF values are higher when DOM is likely more bioavailable (i.e., epipelagic waters). On the other hand, the significant correlation found between eCFs and the peak M/DOC ratio (and peak M/a254, or even peak M/peak T ratios) highlighted the importance of both, quantity (DOM concentration) and quality (fluorescence peak M) of organic compounds as controlling factors in the determination of carbon conversion factors. Taken together, it presumably implies that different DOM molecular groups and their availability in the environment may have an influence in the determination of carbon conversion factors. In this sense, the only significant multiple regression found to explain our eCFs with the physical, chemical and biological variables, linked CF values mainly to peaks M and T (DOM features) and, to a lesser extent, to temperature. It is interesting to note the opposite relation of eCFs with temperature in the multiple regression compared with the bivariate model. Both results are consistent, as these coefficients represent different processes in each correlation. The simple bivariate models represent the direct and complete relation between two variables, while multiple regressions show the correlation with each variable excluding changes due to the others (discriminating processes).

In conclusion, this study showed that empirical leucine-to-carbon conversion factors decreased with depth, showing a wide range of variability throughout the water column for two stations in north-eastern Atlantic waters, resulting in a non-linear dependency of PHP with depth. Our results imply that the use of the theoretical factor of 1.55 kg C mol Leu^−1^ ^6^ in oceanic waters would lead to the underestimation of prokaryotic carbon production in epi- and upper mesopelagic waters, and to its overestimation in bathypelagic waters. In addition, for the first time, we have provided evidence of strong and significant links among eCFs, environmental variables, DOM, and bacterial community composition. An exploratory preliminary multiple regression model is provided as starting point for the estimation of conversion factors in open-ocean waters from relatively simple optical measurements and basic hydrographic observations that can be easily obtained in near real-time. This research illuminates dark-ocean biogeochemistry that is broadly consequential for reconstructing the global carbon cycle.

## Methods

### Sampling strategy

This study was carried out during the MODUPLAN 0814 cruise on board the RV Sarmiento de Gamboa (August 2014), visiting several stations along two perpendicular sections, off the coasts of Galicia (Finisterre section, 43°N, 9°W to 43°N, 14°W) and Cantabria (Santander section, 43°N, 3° 47′W to 45°N, 3° 47′W) (Fig. 1) in the northern Atlantic Ocean. Vertical profiles, from surface to a maximum depth of 5300 m (depending on the bathymetry of each station), as well as seawater sampling, were carried out using a CTD-ADCP-rosette system, provided with oxygen and fluorescence sensors as well as twenty-four 12-L Niskin bottles. Thus, along the cruise we performed: (1) hydrographic characterization (potential temperature, salinity and dissolved oxygen concentration) of the water column (for all the stations; Fig. 1); (2) experiments for the empirical determination of leucine-to-carbon conversion factors (at biological stations: 11, 111 and 115; Fig. 1); (3) determination of bacterial metabolism (leucine incorporation rate to estimate heterotrophic prokaryotic production, at biological stations; Fig. 1), (4) bacterial diversity (stations 11 and 115); and (5) DOM characterization (concentration of DOC and DOM optical properties, at biological stations; Fig. 1).

Since the eCFs were estimated at different depths of the water column, sampling depths were arranged into four layers: epipelagic (< 100 m), upper (100–450 m) and lower (450–1000 m) mesopelagic, and bathypelagic (> 1000 m).

### Experimental setup for determining eCFs

With the aim of determining in situ factors to convert LIRs into carbon bacterial production, dilution experiments were performed at 500, 1000 and 2000 m in stations 11 (Finisterre) and 115 (Santander), and at 50 and 100 m in station 111 (Finisterre). At each station and depth, the water sample was diluted (1:10) with 0.2 µm-filtered (Acropack 1000, Pall) seawater from the same sample and incubated in 2-L polycarbonate bottles in the dark at the corresponding in situ temperature (± 1.5 °C). Subsamples were taken for estimating LIR, and biomass was determined by flow cytometry (see below) at 24-h intervals until bacteria reached the stationary growth phase, after 6–8 days since the beginning of the incubations.

Conversion factors were subsequently calculated following the cumulative method^44^, which estimates the slope of the linear regression between prokaryotic biomass (y-axis) and leucine incorporation (x-axis), accumulated at different time intervals during the time course incubations. One of the limitations of these experiments is that increases in leucine incorporation do not accurately reflect increases in bacterial biomass. This assumption is often not met because of different processes (i.e. grazer influence and/or viral lysis)^45,46^. Hence, to derive resolvable slope values not all time points have been used (i.e. certain data points, where biomass decreases, were excluded).

### Prokaryotic abundance and biomass

Total prokaryotic abundance during the dilution experiments was daily determined on board by flow cytometry, following the method previously described by Gasol et al*.*^47^. Prokaryotic cell counts were detected by their distinct signature in a plot of side scatter vs. green fluorescence using a FACSCalibur flow cytometer (Becton Dickinson). The biovolume of prokaryotic cells was estimated using the calibration obtained by Calvo-Díaz and Morán^48^ relating relative light side scatter (population SSC divided by bead SSC) to cell diameter, assuming spherical shape. Cell biovolume (BBv) was converted into carbon biomass (C; pg cell^−1^) using the allometric relationship of Norland^49^: C (pg cell^−1^) = 0.12 × BBv^0.72^.

### Vertical profiles of LIR and PHP

In situ LIRs were measured using two different methods. The centrifugation method was used for epi- and mesopelagic waters (≤ 1000 m)^15^, whilst the filtration method^15^ was used for bathypelagic samples, because of their typically lower prokaryotic activity. Both methods used ^3^[H]-leucine (160 Ci mmol L^−1^, GE Healthcare) at a final concentration of 5 nmol L^−1^ ^15^. Incubation time and sample volume were adjusted depending on the expected prokaryotic abundance and activity. For the centrifugation method, three replicates of 1.2-mL and two TCA-killed blanks (5% final concentration) were incubated in the dark and at simulated in situ temperature (± 1.5 °C), for 2 to 6 h. The incubations were stopped by adding TCA (5% final concentration). Prokaryotic proteins were precipitated by two successive centrifugation steps (12,000 rpm, 10 min), including one 1-mL 5% TCA wash, following Kirchman et al.^50^ with slight modifications^51^. For the filtration method, 40-mL samples, in duplicate, plus two formaldehyde-killed blanks (2% final concentration) were incubated in the dark at in situ temperature for 6 to 24 h. Subsequently, the incubations were stopped by adding formaldehyde (2% final concentration), filtered through 0.2-µm polycarbonate filters (25 mm of diameter, Millipore), and rinsed twice with 10-mL of 5% ice-cold TCA. Finally, the filters were air dried and transferred to scintillation vials.

For both centrifugated and filtered samples, radioactivity was measured in a scintillation counter (Perkin-Elmer TriCarb 3100TR) after at least 18 h since the addition of the scintillation cocktail (Ultima Gold XR). The disintegrations per minute (DPMs) of the blanks were subtracted from the mean DPMs of the respective samples, and the resultant DPMs were converted into LIRs^51^.

From LIR estimates, PHP (μmol C m^−3^ d^−1^) was calculated as PHP = LIR * CF, where CF is the leucine-to-carbon conversion factor expressed in kg C mol Leu^−1^. The theoretical PHP was determined by applying the theoretical CF proposed by Simon and Azam^6^, 1.55 kg C mol Leu^−1^, while the empirical PHP was determined by applying the in situ eCFs obtained in this study.

### DNA extraction, amplification, sequencing, and bioinformatics

Seawater samples for DNA analyses were collected at each sampling depth by filtering 10–15 L through 0.22-µm Sterivex filters (Millipore). Then, 1.8 mL of lysis buffer (40 Mm EDTA, 50 mMTRIS-HCl, 0.75 M saccharose) was added to the cartridge filter and they were stored at − 80 °C until further analysis. The DNA extraction was performed following the phenol–chloroform extraction method described by Massana et al*.*^52^ with slight modifications^15^. Cell lysis was performed by a 45-min digestion with freshly-made lysozyme (1 mg mL^−1^ final concentration) at 37 °C, followed by a 60-min proteinase K digestion (0.2 mg mL^−1^ final concentration) with sodium dodecyl sulfate (SDS) (10%) at 55 °C. Then, DNA was extracted twice in phenol:chloroform:IAA (25:24:1) and once in chloroform:IAA (24:1). The extracted DNA was concentrated using an Amicon Ultracel 100 k filter unit (Millipore). DNA concentration and purity were quantified according to the A260/A280 ratio using a Nanodrop spectrophotometer (Thermo Scientific, EEUU). Nucleic acid extracts were stored at − 20 °C until further analysis.

The V3 to V4 regions of the 16S rRNA gene were amplified by implementing the polymerase chain reaction (PCR) technique, using the primer pairs 341F and 805R for Bacteria^53^. The 20-µL PCR mixture contained 2 µL of the corresponding primer set (1 µL and 10 µM each), 2 µL 10 × PCR Buffer (Invitrogen), 1.2 µL MgCl_2_ (25 mM), 0.4 µL dNTP (10 mM), 1.25 U Taq polymerase (Platinum, Invitrogen) and 1 µL of DNA templates (approximately 20 ng) and completed with sterilized ultrapure water. PCR amplification was performed by using a Mastercycler (Eppendorf). Cycling conditions for amplification of DNA were 94 °C, 5 min; 30 cycles of 94 °C, 1 min; 57.5 °C, 1 min; 72 °C, 2 min and 72 °C, 10 min)^53^. PCR products were checked for quality control on a 1% (w/v) agarose gel electrophoresis, cleaned and purified with 5-Prime ArchivePure purification kit (Fisher Scientific), and kept at − 20 °C until further analysis.

DNA was analyzed in an Illumina Miseq platform using 2 × 250 bp paired-end approaches. From raw sequence data, primers and spurious sequences were trimmed using *cutadapt* trimming ~ 50 bp. Exact ASVs were differentiated by using *dada2*^54^ implemented in R^55^. The approach is threshold free, inferring exact variants up to one nucleotide of difference using the Q scores in a probability model. This pipeline was implemented through the high-performance supercomputing resources belonging to the Centro Tecnolóxico de Supercomputación de Galicia (CESGA). Sequences were aligned against SILVA 132 16S rRNA database^56^ as reference. Finally, singletons (ASVs found only once in the final ASV table) were excluded, as they have been shown to be likely the result of PCR or sequencing errors^57^. The number of reads per sample ranged from 6,513 to 31,282 in Finisterre and from 9,674 to 25,499 in Santander, with a total of 213,576 reads. The dataset was thus rarefied to the lowest number of reads per sample (6,513 reads) to enable diversity comparisons among samples. ASV richness and diversity metrics were determined implementing the function *estimateR* (*vegan* package,^58^) in R^55^.

### DOC concentration and DOM optical properties

All DOM samples above 200 m were filtered under positive pressure of nitrogen using an acid-clean all-glass system and combusted (450 °C) GFF filters. Water samples for DOC analysis were collected in combusted (450 °C) glass ampoules, and acidified with H_3_PO_4_ to pH < 2. The ampoules were heat-sealed and DOC concentrations were determined with a Shimadzu TOC-V_CSH_ analyzer by high-temperature Pt-catalytic oxidation^59^. Samples were calibrated daily with potassium hydrogen phthalate (99.95–100.05%, p.a., Merck) and the precision of the measurements was 1 µmol C L^−1^. The accuracy of the system was checked with the reference samples supplied by D. A. Hansell (University of Miami, USA).

DOM optical properties were measured on board by pouring directly 5–25 mL of seawater/filtrate in the corresponding optical cell. FDOM was measured using a Perkin Elmer LS55 spectrophotometer, following the method by Nieto-Cid et al*.*^18^, in two excitation/emission wavelengths: (1) 320 nm/410 nm (peak M), and (2) 280 nm/350 nm (peak T). Samples were calibrated against quinine sulfate so the results are given in quinine sulfate units (QSU). On the other hand, the absorption spectra of the chromophoric DOM was obtained by scanning samples between 250 and 700 nm wavelengths, which provide the absorption coefficients a254, a340, a365 and s275-295^60^, using a Beckman Coulter DU800 spectrophotometer equipped with 10 cm quartz cells.

### Statistical analysis

The normality of the variables was tested with the Shapiro–Wilk test^61^. Then, Pearson correlation test^62^, performed in XLSTAT^63^, was used to determine the bivariate correlation between eCFs and the hydrographic features, the DOM optical properties and bacterial diversity. Multiple linear regression models were adjusted using the package STATISTICA by StatSoft.

In order to study the variability of PHP with depth, linear and second order polynomial regression models were fitted to data. The best fitted model was selected according to the lowest value of the Akaike’s Information Criterion (AIC)^64^. Then, one-way analysis of variance (ANOVA) was applied in order to test for significant differences between mean empirical and theoretical PHP. A Tukey’s post hoc test was used to determine which depth layers (epipelagic, upper and lower mesopelagic, and bathypelagic) were significantly different from each other.

To compare bacterial community composition among depth layers, an analysis of similarity (ANOSIM), based on Bray–Curtis dissimilarity, was implemented. Then, a SIMPER analysis determined the main ASVs responsible for the Bray–Curtis dissimilarity between each pair of groups^65^. Both analyses were implemented with the *vegan* package^58^ in R^55^.

All “count zeros” were replaced in the microbial absolute abundance matrix by the Bayesian multiplicative method (function *cmultRepl* in the *zCompositions* package in R), according to Quinn et al*.*^66^. Then, centered log-ratio (CLR) transformation of abundances was performed through the function *clr* (*MASS* package). Finally, linear and second order polynomial regression models were fitted to the relationship between eCFs and the averaged (all depths within each depth layer) (CLR) transformed abundance of ASVs/phylotypes. Again, the best fitted model was selected according to the lower AIC value.

All the statistical analysis were performed in R^55^ unless otherwise specified.

## Supplementary Information

Supplementary Information.
