# Supplementary material for: Empirical leucine-to-carbon conversion factors in north-eastern Atlantic waters (50–2000 m) shaped by bacterial community composition and optical signature of DOM
Source: Sci Rep. 2021 Dec 21;11:24370. doi: 10.1038/s41598-021-03790-y (PMC8692456; doi:10.1038/s41598-021-03790-y)
Supplement: Supplementary file 1 — Supplementary Information. [file 41598_2021_3790_MOESM1_ESM.pdf]

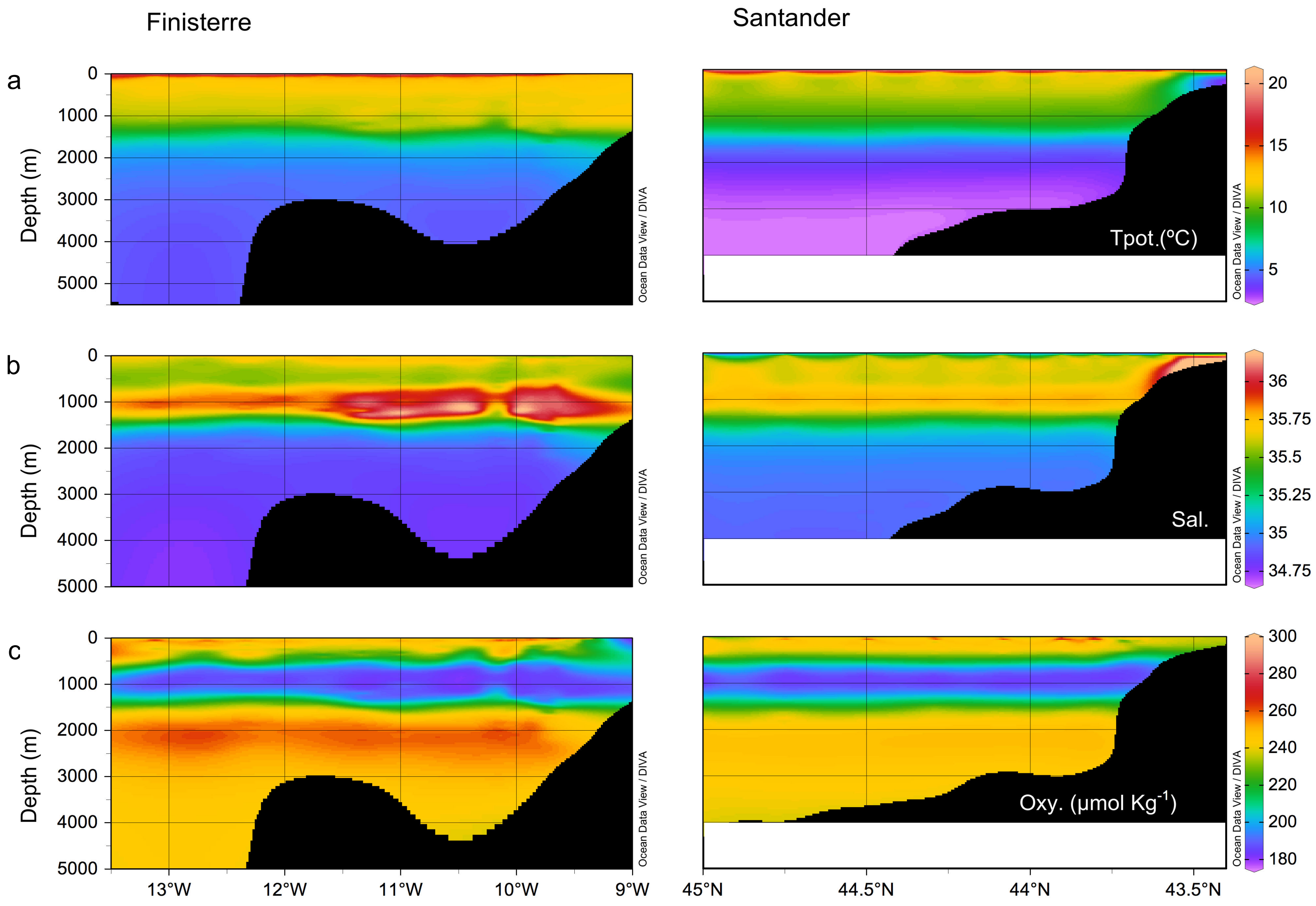


Figure S1. Hydrological characterization of the water column in Finisterre (left panels, 0-5000 m) and Santander (right panels, 0-4000 m): a) potential temperature (Tpot, ºC), b) salinity (Sal), and c) dissolved oxygen concentration (Oxy, µmol $Kg^{-1}$).

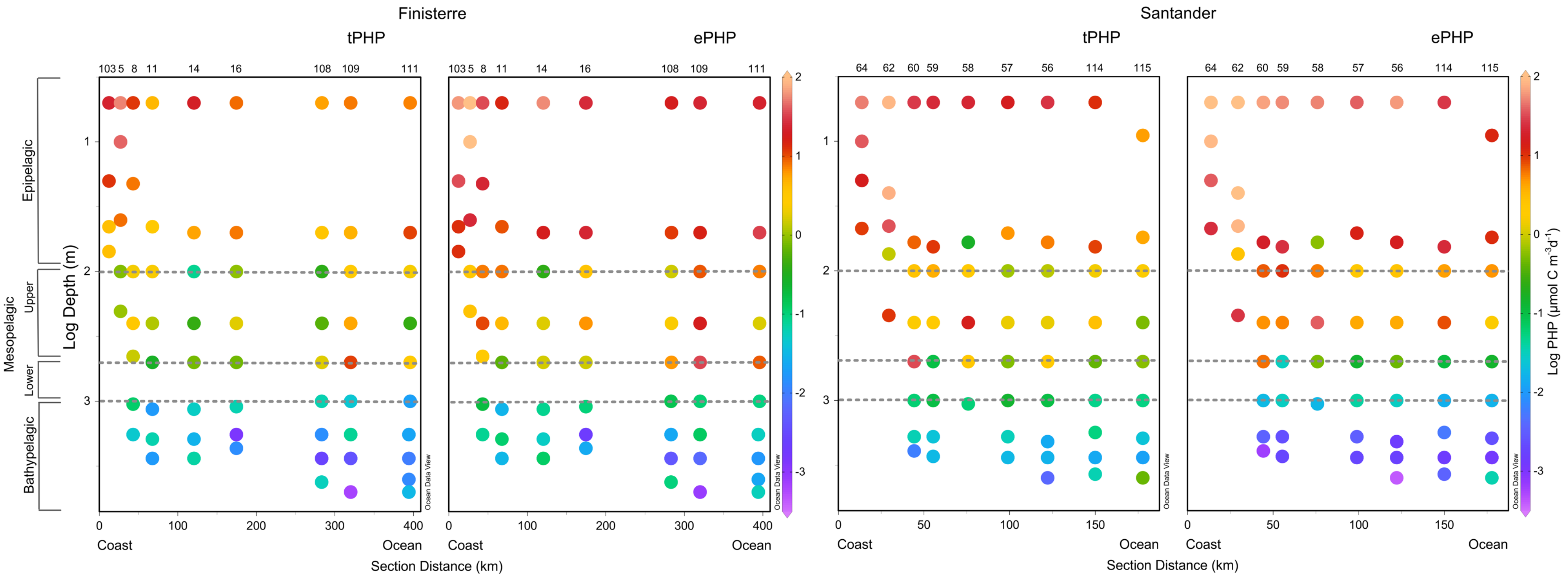


Figure S2. Prokaryotic heterotrophic production (log PHP, μmol C $m^{-3}$ $d^{-1}$) calculated with both the theoretical (tPHP) and the empirical (ePHP) conversion factors, for Finisterre (left) and Santander sections (right). Because epipelagic eCFs were not available for Santander station, those determined at station 111 (Finisterre) were used. To improve visualization, depth is shown in the y-axis in logarithmic scale, and the water column was divided into epipelagic (<100 m), upper mesopelagic (100-450 m), lower mesopelagic (450-1000 m) and bathypelagic (>1000 m) layer. The x-axis indicates the distance of each sampling station (upper numeric labels) from the coast.

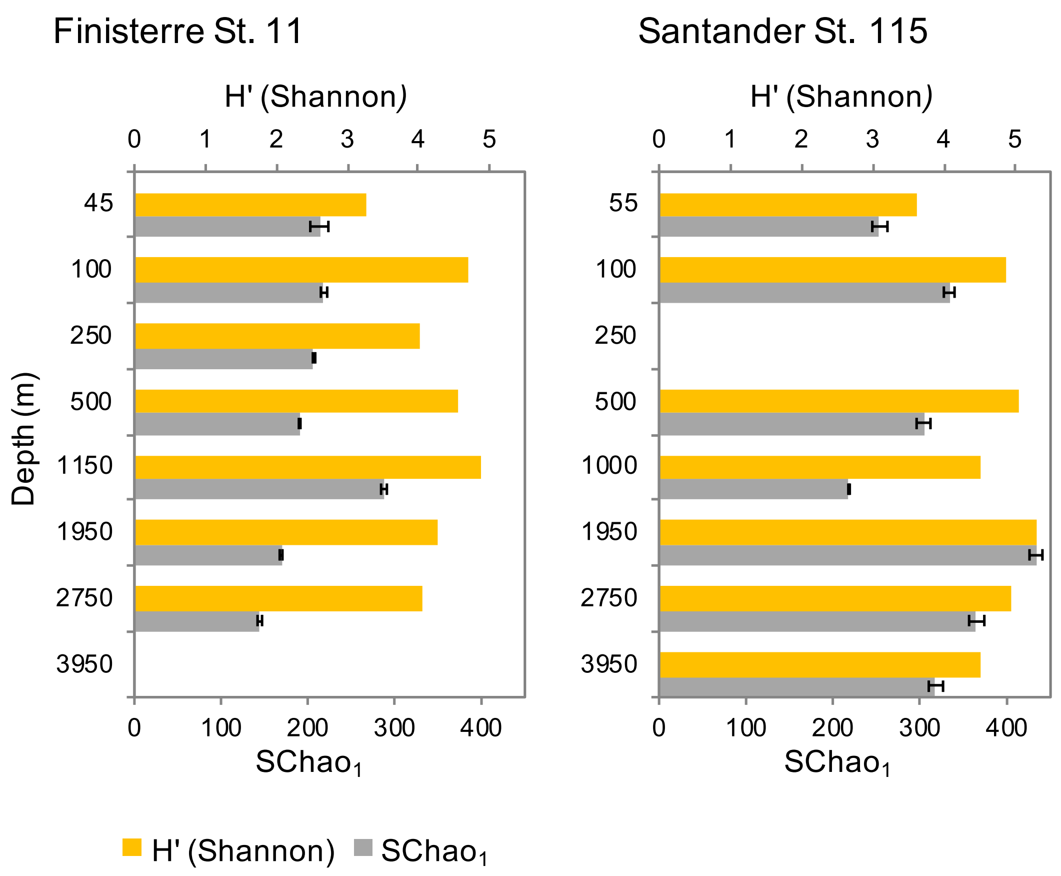


Figure S3. Vertical variability of the Shannon diversity index (H', yellow) and estimated ASV richness ($SChao_1$, grey) in Finisterre (Station 11) and Santander (Station 115). Error bars represent the standard error of $SChao_1$ (s.e.$Chao_1$).

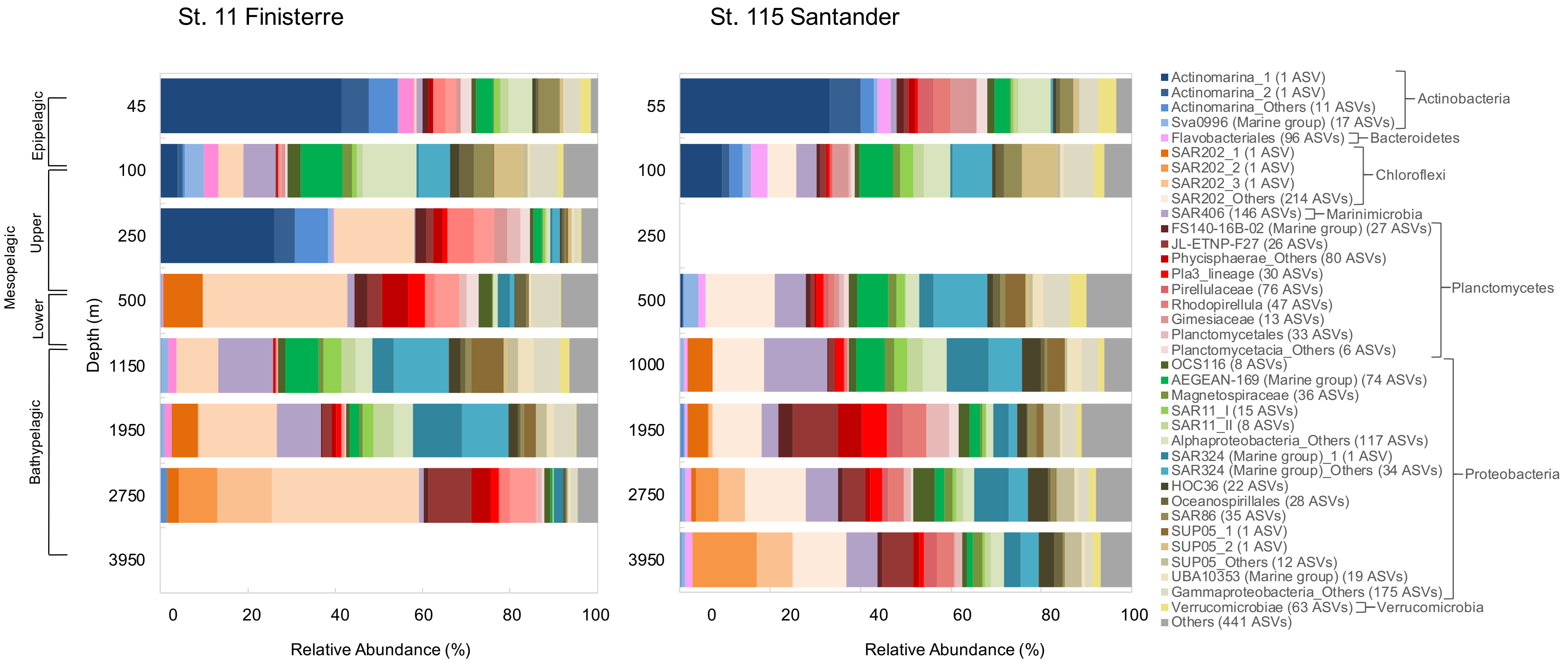


Figure S4. Relative abundance (%) of abundant (relative abundance >1% of total number of reads per station) bacterial ASVs/phylotypes at stations 11 (Finisterre) and 115 (Santander). The number of ASVs pooled within each group was indicated in brackets.

Table S1. Observed and estimated ASV richness (Sobs and $SChao_1$, respectively) and Shannon diversity index (H'), at the different sampling depths in stations 11 (Finisterre) and 115 (Santander). The standard error for $SChao_1$ is given as *s.e.Chao₁*.

| Section | Station | Depth (m) | Sobs | $SChao_1$ | $s.e.Chao_1$ | H' (Shannon) |
|---|---|---|---|---|---|---|
| Finisterre | 11 | 45 | 196 | 213.10 | 10.41 | 3.27 |
| | 11 | 100 | 215 | 218.50 | 3.66 | 4.71 |
| | 11 | 250 | 206 | 207.00 | 1.37 | 4.02 |
| | 11 | 500 | 190 | 190.43 | 0.91 | 4.56 |
| | 11 | 1150 | 283 | 287.62 | 3.33 | 4.90 |
| | 11 | 1950 | 168 | 169.00 | 1.58 | 4.28 |
| | 11 | 2750 | 143 | 144.50 | 2.60 | 4.06 |
| Santander | 115 | 55 | 238 | 253.81 | 8.71 | 3.62 |
| | 115 | 100 | 323 | 333.50 | 6.15 | 4.89 |
| | 115 | 500 | 292 | 304.00 | 7.96 | 5.06 |
| | 115 | 1000 | 218 | 218.38 | 0.83 | 4.53 |
| | 115 | 1950 | 416 | 433.23 | 7.43 | 5.31 |
| | 115 | 2750 | 347 | 365.13 | 8.75 | 4.94 |
| | 115 | 3950 | 301 | 318.22 | 8.21 | 4.51 |

Table S2. SIMPER analysis identifying those ASVs/phylotypes which cumulatively accounted for 70% of compositional differences in bacterial communities between the compared depth layers.

| | Depth layers | | | | | |
|---|---|---|---|---|---|---|
| **ASV/phylotype** | **Epipelagic vs Upper Mesopelagic** | **Epipelagic vs Lower Mesopelagic** | **Epipelagic vs Bathypelagic** | **Upper Mesopelagic vs Lower Mesopelagic** | **Upper Mesopelagic vs Bathypelagic** | **Lower Mesopelagic vs Bathypelagic** |
| Actinomarina_1 | 18.32 | 19.68 | 17.18 | 13.20 | - | 13.26 |
| SAR202_Others | 13.51 | 14.74 | 10.11 | 10.83 | 13.09 | 6.63 |
| SAR324 (Marine group)_Others | 5.74 | 4.56 | 3.99 | 5.32 | 5.43 | 5.52 |
| SAR406 | 3.31 | 4.91 | 3.67 | 6.71 | 7.16 | 4.82 |
| AEGEAN-169 (Marine group) | 3.18 | 3.29 | 3.26 | 3.47 | 3.83 | 2.98 |
| SAR324 (Marine group)_1 | - | 3.00 | 4.17 | 4.30 | 4.22 | 3.71 |
| SAR202_1 | - | 5.72 | - | 7.37 | 5.82 | - |
| Actinomarina_Others | 3.54 | 2.60 | 2.29 | 4.00 | - | 3.70 |
| Alphaproteobacteria_Others | 5.89 | 3.69 | 4.12 | - | - | - |
| Actinomarina_2 | 3.27 | 3.05 | 3.08 | 2.40 | - | 2.41 |
| JL-ETNP-F27 | - | - | 4.10 | - | 5.04 | 4.91 |
| SAR202_2 | - | - | 3.70 | - | 5.44 | 4.74 |
| SAR202_3 | - | - | 3.59 | - | 5.27 | 4.59 |
| Gammaproteobacteria_Others | 2.58 | - | - | 2.75 | 2.87 | 2.40 |
| SUP05_2 | 3.12 | 3.08 | 3.01 | - | - | - |
| SAR86 | 3.31 | 2.80 | 2.44 | - | - | - |
| Pirellulaceae | 2.79 | - | - | 2.85 | - | 2.79 |
| Rhodopirellula | - | - | - | 2.67 | 3.20 | - |
| SUP05_Others | - | - | - | - | 2.90 | 2.47 |
| SUP05_1 | - | - | - | - | 2.81 | 2.62 |
| Actinomarina_Others | - | - | - | - | 3.25 | - |
| Phycisphaerae_Others | - | - | - | 2.67 | - | - |
| Others | 3.77 | 2.33 | 2.48 | 3.18 | - | 3.36 |
| **TOTAL** | **72.32** | **70.75** | **71.20** | **71.81** | **70.33** | **71.02** |

Table S3. Linear and quadratic models fitted to the relationship between the centered log-ratio (CLR) transformed abundance of each ASV/phylotype and eCFs. $R^2$, the coefficient of determination, shows the percentage of variation of the average abundance explained by the eCFs; $P$ is the significance level. Akaike's Information Criterion (AIC) in bold correspond to the best fitted model for each ASV/phylotype.

| ASV/phylotype | LINEAR | | | QUADRATIC | | |
|---|---|---|---|---|---|---|
| | $R^2$ | P | AIC | $R^2$ | P | AIC |
| Actinomarina_1 | 0.67 | 0.0071 | 35.38 | 0.76 | 0.0133 | **34.35** |
| Actinomarina_2 | 0.75 | 0.0026 | 32.92 | 0.93 | 0.0003 | **23.00** |
| Actinomarina_Others | 0.22 | 0.2054 | 40.29 | 0.55 | 0.0938 | **37.40** |
| Sva0996 (Marine group) | 0.00 | 0.9586 | **31.42** | 0.07 | 0.8145 | 32.81 |
| Flavobacteriales | 0.22 | 0.2085 | **28.85** | 0.25 | 0.4206 | 30.43 |
| SAR202_1 | 0.17 | 0.2729 | **49.44** | 0.19 | 0.5384 | 51.24 |
| SAR202_2 | 0.16 | 0.2832 | 31.39 | 0.35 | 0.2721 | **31.07** |
| SAR202_3 | 0.14 | 0.3267 | **36.60** | 0.15 | 0.6104 | 38.45 |
| SAR202_Others | 0.37 | 0.0835 | 27.29 | 0.57 | 0.0788 | **25.79** |
| SAR406 | 0.20 | 0.2305 | **26.33** | 0.22 | 0.4695 | 28.05 |
| FS140-16B-02 (Marine group) | 0.18 | 0.2556 | **40.15** | 0.18 | 0.5511 | 42.14 |
| JL-ETNP-F27 | 0.19 | 0.2408 | **44.10** | 0.24 | 0.4457 | 45.57 |
| Phycisphaerae_Others | 0.00 | 0.8585 | **39.43** | 0.04 | 0.8732 | 41.07 |
| Pla3_lineage | 0.75 | 0.0024 | 31.74 | 0.81 | 0.0069 | **31.45** |
| Pirellulaceae | 0.00 | 0.8767 | 42.90 | 0.32 | 0.3135 | **41.45** |
| Rhodopirellula | 0.00 | 0.9162 | **34.79** | 0.09 | 0.7530 | 35.96 |
| Gimesiaceae | 0.26 | 0.1621 | 39.89 | 0.61 | 0.0595 | **36.12** |
| Planctomycetales | 0.46 | 0.0431 | **40.02** | 0.48 | 0.1435 | 41.82 |
| Planctomycetacia_Others | 0.05 | 0.5707 | **42.96** | 0.23 | 0.4493 | 43.00 |
| OCS116 | 0.00 | 0.9386 | **16.57** | 0.04 | 0.8894 | 18.23 |
| AEGEAN-169 (Marine group) | 0.00 | 0.8962 | 42.91 | 0.21 | 0.4878 | **42.78** |
| Magnetospiraceae | 0.15 | 0.3077 | **28.10** | 0.16 | 0.5897 | 29.95 |
| SAR11_1 | 0.04 | 0.5858 | **30.98** | 0.05 | 0.8685 | 32.97 |
| SAR11_2 | 0.01 | 0.8127 | **41.85** | 0.01 | 0.9711 | 43.84 |
| Alphaproteobacteria_Others | 0.24 | 0.1836 | **23.21** | 0.32 | 0.3094 | 24.13 |

| | | | | | | |
|---|---|---|---|---|---|---|
| SAR324 (Marine group)_1 | 0.60 | 0.0137 | 34.90 | 0.76 | 0.0140 | **32.45** |
| SAR324 (Marine group)_Others | 0.23 | 0.1902 | **35.87** | 0.30 | 0.3496 | 37.08 |
| HOC36 | 0.18 | 0.2603 | **25.47** | 0.21 | 0.4978 | 27.12 |
| Oceanospirillales | 0.10 | 0.4139 | **23.39** | 0.12 | 0.6865 | 25.19 |
| SAR86 | 0.23 | 0.1895 | 28.21 | 0.58 | 0.0764 | **24.87** |
| SUP05_1 | 0.57 | 0.0188 | **35.29** | 0.65 | 0.0428 | 35.41 |
| SUP05_2 | 0.28 | 0.1392 | **43.21** | 0.32 | 0.3119 | 44.73 |
| SUP05_Others | 0.11 | 0.3923 | **39.56** | 0.13 | 0.6475 | 41.26 |
| UBA10353 (Marine group) | 0.47 | 0.0414 | **32.82** | 0.55 | 0.0922 | 33.39 |
| Gammaproteobacteria_Others | 0.04 | 0.5864 | 7.81 | 0.39 | 0.2238 | **5.73** |
| Verrucomicrobiae | 0.00 | 0.9058 | 32.85 | 0.24 | 0.4459 | **32.44** |
| Others | 0.33 | 0.1037 | **17.01** | 0.33 | 0.2962 | 19.01 |
